# Supplementary material for: PqsE has a conserved sequence, yet a variable impact in Pseudomonas aeruginosa
Source: J Bacteriol. 2025 Oct 17;207(11):e00402-25. doi: 10.1128/jb.00402-25 (PMC12632258; doi:10.1128/jb.00402-25)
Supplement: Supplemental figures and tables — Figures S1 to S3 and Tables S1 to S10. [file jb.00402-25-s0001.pdf]

Supplementary data

PqsE has a Conserved Sequence, yet a Variable Impact in *Pseudomonas aeruginosa*

Mylène C. Trottier[1], Marie-Christine Groleau[1], Jeff Gauthier[2], Antony T. Vincent[3], Roger C. Levesque[2], Eric Déziel[1*]

[1]Centre Armand-Frappier Santé Biotechnologie, Institut National de la Recherche Scientifique (INRS), Laval, Québec, H7V 1B7, Canada

[2]Institut de biologie intégrative et des systèmes (IBIS), Faculté de médecine, Université Laval, Québec City, G1V 0A6, Canada

[3]Département des sciences animales, Faculté des sciences de l'agriculture et de l'alimentation, Université Laval, Québec City, Québec, G1V 0A6, Canada

*Correspondence to Eric Déziel : eric.deziel@inrs.ca

Table S1: List of strains used in this study

Strains	Lab ID (#)	Characteristics	Source
		P. aeruginosa	
PA14	ED14	Clinical isolate UCBPP-PA14	(1)
PA14Δ*pqsE*	ED36	PA14 derivate; unmarked in-frame *pqsE* deletion	(2)
PA14Δ$pqsA_{np}$	ED83	PA14 derivate; unmarked in-frame nonpolar *pqsA* deletion	(3)
PAO1-N	ED4866	PAO1 from the Nottingham collection	G. Rampioni
PAO1-NΔ*pqsE*	ED4867	PAO1-N derivate; unmarked in-frame *pqsE* deletion	(4)
39016	ED4372	Keratitis isolate	(5)
39016Δ*pqsE*	ED4861	39016 derivate; unmarked in-frame *pqsE* deletion	This study
A22	ED4350	Wound isolate	(6)
A22Δ*pqsE*	ED4697	A22 derivate; unmarked in-frame *pqsE* deletion	This study
E90	ED3900	Cystic fibrosis isolate	(7)
E90Δ*pqsE*	ED4856	E90 derivate; unmarked in-frame *pqsE* deletion	This study
JJ692	ED4337	Urinary tract infection isolate	(8)
JJ692Δ*pqsE*	ED4859	JJ692 derivate; unmarked in-frame *pqsE* deletion	This study
PA-W9	ED4357	Leg Ulcer isolate	(5)
PA-W9Δ*pqsE*	ED4778	PA-W9 derivate; unmarked in-frame *pqsE* deletion	This study
SMC1596	ED4672	Cystic fibrosis isolate	(9)
SMC1596Δ*pqsE*	ED4862	SMC1596 derivate; unmarked in-frame *pqsE* deletion	This study
PA-CL512	ED4909	Hospital sink isolate	(10)
PA-CL512Δ*pqsE*	ED4739	PA-CL512 derivate; unmarked in-frame *pqsE* deletion	This study
PA-CL513	ED4910	Hospital sink isolate	(10)
PA-CL513Δ*pqsE*	ED4696	PA-CL513 derivate; unmarked in-frame *pqsE* deletion	This study
PA-CL521b	ED4911	Hospital sink isolate	(10)
PA-CL521bΔ*pqsE*	ED4779	PA-CL521b derivate; unmarked in-frame *pqsE* deletion	This study
PG201 (Rsan-ver)	ED124	Soil isolate	(11)
PG201Δ*pqsE*	ED4863	PG201 derivate; unmarked in-frame *pqsE* deletion	This study
		E. coli	
χ7213	ED743	*thr-1 leuB6 fhuA21 lacY1 glnV44 recA1 ΔasdA4 Δ(zhf-2::Tn10) thi-1 RP4-2-Tc::Mu [λ pir]*	Lab collection

Table S2: Single nucleotide polymorphisms (SNPs) in DNA sequence of the *pqsE* gene in twelve strains of *P. aeruginosa*

Strain	Position of nucleotide in DNA sequence			
	75	174	516	768
**PA14**	T	T	A	G
**PAO1**	C	C	A	A
**39016**	T	T	A	G
**A22**	C	C	A	A
**E90**	C	C	A	A
**JJ692**	T	T	A	G
**PA-CL512**	C	C	A	A
**PA-CL513**	T	T	A	G
**PA-CL521b**	C	C	A	A
**PA-W9**	C	C	A	A
**PG201**	C	C	A	A
**SMC1596**	C	C	C	A

```
PA14        MLRLSAPGQLDDDLCLLGDVQVPVFLLRLGEASWALVEGGISRDAELVWADLCRWVADPS
PAO1-N      MLRLSAPGQLDDDLCLLGDVQVPVFLLRLGEASWALVEGGISRDAELVWADLCRWVADPS
39016       MLRLSAPGQLDDDLCLLGDVQVPVFLLRLGEASWALVEGGISRDAELVWADLCRWVADPS
A22         MLRLSAPGQLDDDLCLLGDVQVPVFLLRLGEASWALVEGGISRDAELVWADLCRWVADPS
CL512       MLRLSAPGQLDDDLCLLGDVQVPVFLLRLGEASWALVEGGISRDAELVWADLCRWVADPS
CL513       MLRLSAPGQLDDDLCLLGDVQVPVFLLRLGEASWALVEGGISRDAELVWADLCRWVADPS
CL521b      MLRLSAPGQLDDDLCLLGDVQVPVFLLRLGEASWALVEGGISRDAELVWADLCRWVADPS
E90         MLRLSAPGQLDDDLCLLGDVQVPVFLLRLGEASWALVEGGISRDAELVWADLCRWVADPS
JJ692       MLRLSAPGQLDDDLCLLGDVQVPVFLLRLGEASWALVEGGISRDAELVWADLCRWVADPS
PA-W9       MLRLSAPGQLDDDLCLLGDVQVPVFLLRLGEASWALVEGGISRDAELVWADLCRWVADPS
PG201       MLRLSAPGQLDDDLCLLGDVQVPVFLLRLGEASWALVEGGISRDAELVWADLCRWVADPS
SMC1596     MLRLSAPGQLDDDLCLLGDVQVPVFLLRLGEASWALVEGGISRDAELVWADLCRWVADPS
            ************************************************************

PA14        QVHYWLITHKHYDHCGLLPYLCPRLPNVQVLASERTCQAWKSESAVRVVERLNRQLLRAE
PAO1-N      QVHYWLITHKHYDHCGLLPYLCPRLPNVQVLASERTCQAWKSESAVRVVERLNRQLLRAE
39016       QVHYWLITHKHYDHCGLLPYLCPRLPNVQVLASERTCQAWKSESAVRVVERLNRQLLRAE
A22         QVHYWLITHKHYDHCGLLPYLCPRLPNVQVLASERTCQAWKSESAVRVVERLNRQLLRAE
CL512       QVHYWLITHKHYDHCGLLPYLCPRLPNVQVLASERTCQAWKSESAVRVVERLNRQLLRAE
CL513       QVHYWLITHKHYDHCGLLPYLCPRLPNVQVLASERTCQAWKSESAVRVVERLNRQLLRAE
CL521b      QVHYWLITHKHYDHCGLLPYLCPRLPNVQVLASERTCQAWKSESAVRVVERLNRQLLRAE
E90         QVHYWLITHKHYDHCGLLPYLCPRLPNVQVLASERTCQAWKSESAVRVVERLNRQLLRAE
JJ692       QVHYWLITHKHYDHCGLLPYLCPRLPNVQVLASERTCQAWKSESAVRVVERLNRQLLRAE
PA-W9       QVHYWLITHKHYDHCGLLPYLCPRLPNVQVLASERTCQAWKSESAVRVVERLNRQLLRAE
PG201       QVHYWLITHKHYDHCGLLPYLCPRLPNVQVLASERTCQAWKSESAVRVVERLNRQLLRAE
SMC1596     QVHYWLITHKHYDHCGLLPYLCPRLPNVQVLASERTCQAWKSESAVRVVERLNRQLLRAE
            ************************************************************

PA14        QRLPEACAWDALPVRAVADGEWLELGPRHRLQVIEAHGHSDDHVVFYDVRRRRLFCGDAL
PAO1-N      QRLPEACAWDALPVRAVADGEWLELGPRHRLQVIEAHGHSDDHVVFYDVRRRRLFCGDAL
39016       QRLPEACAWDALPVRAVADGEWLELGPRHRLQVIEAHGHSDDHVVFYDVRRRRLFCGDAL
A22         QRLPEACAWDALPVRAVADGEWLELGPRHRLQVIEAHGHSDDHVVFYDVRRRRLFCGDAL
CL512       QRLPEACAWDALPVRAVADGEWLELGPRHRLQVIEAHGHSDDHVVFYDVRRRRLFCGDAL
CL513       QRLPEACAWDALPVRAVADGEWLELGPRHRLQVIEAHGHSDDHVVFYDVRRRRLFCGDAL
CL521b      QRLPEACAWDALPVRAVADGEWLELGPRHRLQVIEAHGHSDDHVVFYDVRRRRLFCGDAL
E90         QRLPEACAWDALPVRAVADGEWLELGPRHRLQVIEAHGHSDDHVVFYDVRRRRLFCGDAL
JJ692       QRLPEACAWDALPVRAVADGEWLELGPRHRLQVIEAHGHSDDHVVFYDVRRRRLFCGDAL
PA-W9       QRLPEACAWDALPVRAVADGEWLELGPRHRLQVIEAHGHSDDHVVFYDVRRRRLFCGDAL
PG201       QRLPEACAWDALPVRAVADGEWLELGPRHRLQVIEAHGHSDDHVVFYDVRRRRLFCGDAL
SMC1596     QRLPEACAWDALPVRAVADGEWLELGPRHRLQVIEAHGHSDDHVVFYDVRRRRLFCGDAL
            ************************************************************
```

```
PA14        GEFDEAEGVWRPLVFDDMEAYLESLERLQRLPTLLQLIPGHGGLLRGRLAADGAESAYTE
PAO1-N      GEFDEAEGVWRPLVFDDMEAYLESLERLQRLPTLLQLIPGHGGLLRGRLAADGAESAYTE
39016       GEFDEAEGVWRPLVFDDMEAYLESLERLQRLPTLLQLIPGHGGLLRGRLAADGAESAYTE
A22         GEFDEAEGVWRPLVFDDMEAYLESLERLQRLPTLLQLIPGHGGLLRGRLAADGAESAYTE
CL512       GEFDEAEGVWRPLVFDDMEAYLESLERLQRLPTLLQLIPGHGGLLRGRLAADGAESAYTE
CL513       GEFDEAEGVWRPLVFDDMEAYLESLERLQRLPTLLQLIPGHGGLLRGRLAADGAESAYTE
CL521b      GEFDEAEGVWRPLVFDDMEAYLESLERLQRLPTLLQLIPGHGGLLRGRLAADGAESAYTE
E90         GEFDEAEGVWRPLVFDDMEAYLESLERLQRLPTLLQLIPGHGGLLRGRLAADGAESAYTE
JJ692       GEFDEAEGVWRPLVFDDMEAYLESLERLQRLPTLLQLIPGHGGLLRGRLAADGAESAYTE
PA-W9       GEFDEAEGVWRPLVFDDMEAYLESLERLQRLPTLLQLIPGHGGLLRGRLAADGAESAYTE
PG201       GEFDEAEGVWRPLVFDDMEAYLESLERLQRLPTLLQLIPGHGGLLRGRLAADGAESAYTE
SMC1596     GEFDEAEGVWRPLVFDDMEAYLESLERLQRLPTLLQLIPGHGGLLRGRLAADGAESAYTE
            ************************************************************

PA14        CLRLCRRLLWRQSMGESLDELSEELHRAWGGQSVDFLPGELHLGSMRRMLEILSRQALPLD
PAO1-N      CLRLCRRLLWRQSMGESLDELSEELHRAWGGQSVDFLPGELHLGSMRRMLEILSRQALPLD
39016       CLRLCRRLLWRQSMGESLDELSEELHRAWGGQSVDFLPGELHLGSMRRMLEILSRQALPLD
A22         CLRLCRRLLWRQSMGESLDELSEELHRAWGGQSVDFLPGELHLGSMRRMLEILSRQALPLD
CL512       CLRLCRRLLWRQSMGESLDELSEELHRAWGGQSVDFLPGELHLGSMRRMLEILSRQALPLD
CL513       CLRLCRRLLWRQSMGESLDELSEELHRAWGGQSVDFLPGELHLGSMRRMLEILSRQALPLD
CL521b      CLRLCRRLLWRQSMGESLDELSEELHRAWGGQSVDFLPGELHLGSMRRMLEILSRQALPLD
E90         CLRLCRRLLWRQSMGESLDELSEELHRAWGGQSVDFLPGELHLGSMRRMLEILSRQALPLD
JJ692       CLRLCRRLLWRQSMGESLDELSEELHRAWGGQSVDFLPGELHLGSMRRMLEILSRQALPLD
PA-W9       CLRLCRRLLWRQSMGESLDELSEELHRAWGGQSVDFLPGELHLGSMRRMLEILSRQALPLD
PG201       CLRLCRRLLWRQSMGESLDELSEELHRAWGGQSVDFLPGELHLGSMRRMLEILSRQALPLD
SMC1596     CLRLCRRLLWRQSMGESLDELSEELHRAWGGQSVDFLPGELHLGSMRRMLEILSRQALPLD
            *************************************************************
```

Figure S1 : Alignment of predicted PqsE protein amino acid sequence in twelve selected *P. aeruginosa* strains. Amino acid sequences of the predicted PqsE protein from selected *P. aeruginosa* strains, including clinical, environmental, and reference strains (PA14 and PAO1), are aligned using MAFFT v7.511 (12). Sequences were obtained from available genomic data (5) and annotated with Prokka v1.13 (13). Identical residues across all strains are marked with an asterisk (*).

Table S3: Single nucleotide polymorphisms (SNPs) in DNA sequence of the *rhlR* gene in twelve strains of *P. aeruginosa*

Strain	Position of nucleotide in DNA sequence										
	**138**	**147**	**291**	**363**	**364**	**453**	**567**	**591**	**621**	**696**	**717**
**PA14**	C	C	C	C	C	C	G	T	C	C	C
**PAO1**	C	C	C	C	T	C	G	T	C	C	T
**39016**	C	C	C	C	C	C	C	T	C	T	C
**A22**	C	C	T	C	C	C	G	C	C	C	C
**E90**	C	T	C	C	C	A	G	C	T	C	C
**JJ692**	C	C	C	C	C	C	G	T	C	T	C
**PA-CL512**	C	C	C	C	C	A	G	C	T	C	C
**PA-CL513**	C	C	C	C	C	C	G	T	C	C	C
**PA-CL521b**	C	T	C	C	C	A	G	C	T	C	C
**PA-W9**	T	T	C	C	C	A	G	C	C	C	C
**PG201**	C	T	C	T	T	A	G	C	T	C	C
**SMC1596**	T	T	C	C	T	A	G	C	T	C	C

```
PA14       MRNDGGFLLWWDGLRSEMQPIHDSQGVFAVLEKEVRRLGFDYYAYGVRHTIPFTRPKTEV
PAO1-N     MRNDGGFLLWWDGLRSEMQPIHDSQGVFAVLEKEVRRLGFDYYAYGVRHTIPFTRPKTEV
39016      MRNDGGFLLWWDGLRSEMQPIHDSQGVFAVLEKEVRRLGFDYYAYGVRHTIPFTRPKTEV
A22        MRNDGGFLLWWDGLRSEMQPIHDSQGVFAVLEKEVRRLGFDYYAYGVRHTIPFTRPKTEV
CL512      MRNDGGFLLWWDGLRSEMQPIHDSQGVFAVLEKEVRRLGFDYYAYGVRHTIPFTRPKTEV
CL513      MRNDGGFLLWWDGLRSEMQPIHDSQGVFAVLEKEVRRLGFDYYAYGVRHTIPFTRPKTEV
CL521b     MRNDGGFLLWWDGLRSEMQPIHDSQGVFAVLEKEVRRLGFDYYAYGVRHTIPFTRPKTEV
E90        MRNDGGFLLWWDGLRSEMQPIHDSQGVFAVLEKEVRRLGFDYYAYGVRHTIPFTRPKTEV
JJ692      MRNDGGFLLWWDGLRSEMQPIHDSQGVFAVLEKEVRRLGFDYYAYGVRHTIPFTRPKTEV
PAW9       MRNDGGFLLWWDGLRSEMQPIHDSQGVFAVLEKEVRRLGFDYYAYGVRHTIPFTRPKTEV
PG201      MRNDGGFLLWWDGLRSEMQPIHDSQGVFAVLEKEVRRLGFDYYAYGVRHTIPFTRPKTEV
SMC1596    MRNDGGFLLWWDGLRSEMQPIHDSQGVFAVLEKEVRRLGFDYYAYGVRHTIPFTRPKTEV
           ************************************************************

PA14       HGTYPKAWLERYQMQNYGAVDPAILNGLRSSEMVVWSDSLFDQSRMLWNEARDWGLCVGA
PAO1-N     HGTYPKAWLERYQMQNYGAVDPAILNGLRSSEMVVWSDSLFDQSRMLWNEARDWGLCVGA
39016      HGTYPKAWLERYQMQNYGAVDPAILNGLRSSEMVVWSDSLFDQSRMLWNEARDWGLCVGA
A22        HGTYPKAWLERYQMQNYGAVDPAILNGLRSSEMVVWSDSLFDQSRMLWNEARDWGLCVGA
CL512      HGTYPKAWLERYQMQNYGAVDPAILNGLRSSEMVVWSDSLFDQSRMLWNEARDWGLCVGA
CL513      HGTYPKAWLERYQMQNYGAVDPAILNGLRSSEMVVWSDSLFDQSRMLWNEARDWGLCVGA
CL521b     HGTYPKAWLERYQMQNYGAVDPAILNGLRSSEMVVWSDSLFDQSRMLWNEARDWGLCVGA
E90        HGTYPKAWLERYQMQNYGAVDPAILNGLRSSEMVVWSDSLFDQSRMLWNEARDWGLCVGA
JJ692      HGTYPKAWLERYQMQNYGAVDPAILNGLRSSEMVVWSDSLFDQSRMLWNEARDWGLCVGA
PAW9       HGTYPKAWLERYQMQNYGAVDPAILNGLRSSEMVVWSDSLFDQSRMLWNEARDWGLCVGA
PG201      HGTYPKAWLERYQMQNYGAVDPAILNGLRSSEMVVWSDSLFDQSRMLWNEARDWGLCVGA
SMC1596    HGTYPKAWLERYQMQNYGAVDPAILNGLRSSEMVVWSDSLFDQSRMLWNEARDWGLCVGA
           ************************************************************

PA14       TLPIRAPNNLLSVLSVARDQQNISSFEREEIRLRLRCMIELLTQKLTDLEHPMLMSNPVC
PAO1-N     TLPIRAPNNLLSVLSVARDQQNISSFEREEIRLRLRCMIELLTQKLTDLEHPMLMSNPVC
39016      TLPIRAPNNLLSVLSVARDQQNISSFEREEIRLRLRCMIELLTQKLTDLEHPMLMSNPVC
A22        TLPIRAPNNLLSVLSVARDQQNISSFEREEIRLRLRCMIELLTQKLTDLEHPMLMSNPVC
CL512      TLPIRAPNNLLSVLSVARDQQNISSFEREEIRLRLRCMIELLTQKLTDLEHPMLMSNPVC
CL513      TLPIRAPNNLLSVLSVARDQQNISSFEREEIRLRLRCMIELLTQKLTDLEHPMLMSNPVC
CL521b     TLPIRAPNNLLSVLSVARDQQNISSFEREEIRLRLRCMIELLTQKLTDLEHPMLMSNPVC
E90        TLPIRAPNNLLSVLSVARDQQNISSFEREEIRLRLRCMIELLTQKLTDLEHPMLMSNPVC
JJ692      TLPIRAPNNLLSVLSVARDQQNISSFEREEIRLRLRCMIELLTQKLTDLEHPMLMSNPVC
PAW9       TLPIRAPNNLLSVLSVARDQQNISSFEREEIRLRLRCMIELLTQKLTDLEHPMLMSNPVC
PG201      TLPIRAPNNLLSVLSVARDQQNISSFEREEIRLRLRCMIELLTQKLTDLEHPMLMSNPVC
SMC1596    TLPIRAPNNLLSVLSVARDQQNISSFEREEIRLRLRCMIELLTQKLTDLEHPMLMSNPVC
           ************************************************************
```

```
PA14        LSHREREILQWTADGKSSGEIAIILSISESTVNFHHKNIQKKFDAPNKTLAAAYAAALGLI
PAO1-N      LSHREREILQWTADGKSSGEIAIILSISESTVNFHHKNIQKKFDAPNKTLAAAYAAALGLI
39016       LSHREREILQWTADGKSSGEIAIILSISESTVNFHHKNIQKKFDAPNKTLAAAYAAALGLI
A22         LSHREREILQWTADGKSSGEIAIILSISESTVNFHHKNIQKKFDAPNKTLAAAYAAALGLI
CL512       LSHREREILQWTADGKSSGEIAIILSISESTVNFHHKNIQKKFDAPNKTLAAAYAAALGLI
CL513       LSHREREILQWTADGKSSGEIAIILSISESTVNFHHKNIQKKFDAPNKTLAAAYAAALGLI
CL521b      LSHREREILQWTADGKSSGEIAIILSISESTVNFHHKNIQKKFDAPNKTLAAAYAAALGLI
E90         LSHREREILQWTADGKSSGEIAIILSISESTVNFHHKNIQKKFDAPNKTLAAAYAAALGLI
JJ692       LSHREREILQWTADGKSSGEIAIILSISESTVNFHHKNIQKKFDAPNKTLAAAYAAALGLI
PAW9        LSHREREILQWTADGKSSGEIAIILSISESTVNFHHKNIQKKFDAPNKTLAAAYAAALGLI
PG201       LSHREREILQWTADGKSSGEIAIILSISESTVNFHHKNIQKKFDAPNKTLAAAYAAALGLI
SMC1596     LSHREREILQWTADGKSSGEIAIILSISESTVNFHHKNIQKKFDAPNKTLAAAYAAALGLI
            *************************************************************
```

Figure S2: Alignment of predicted RhlR protein amino acid sequence in twelve *P. aeruginosa* strains. Amino acid sequences of the predicted RhlR protein from selected *P. aeruginosa* strains, including clinical, environmental, and reference strains (PA14 and PAO1), are aligned using MAFFT v7.511 (12). Sequences were obtained from available genomic data (5) and annotated with Prokka v1.13 (13). Identical residues across all strains are marked with an asterisk (*).

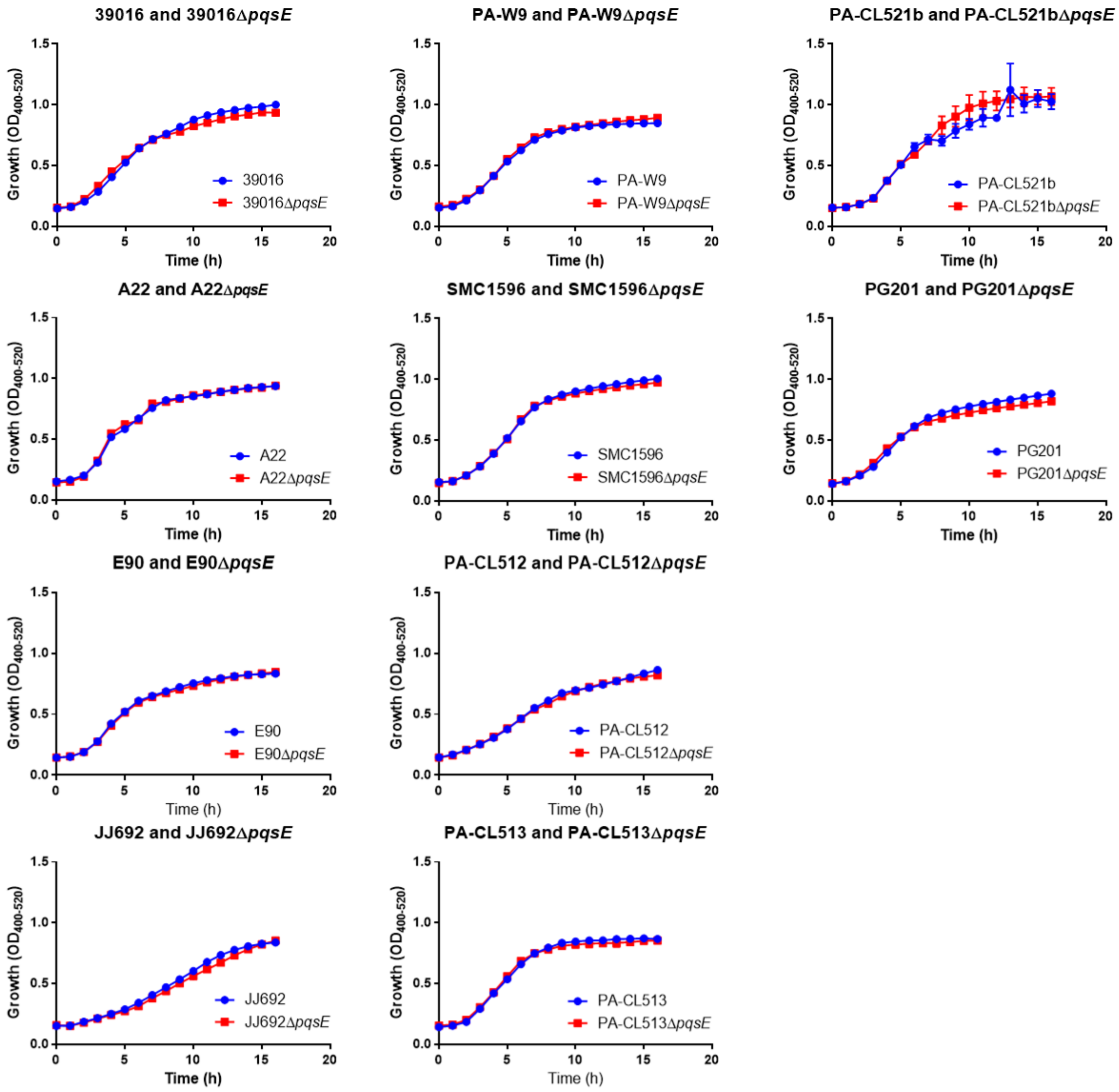

Figure S3: Growth curves of *P. aeruginosa* isolates and their isogenic Δ*pqsE* mutants. Overnight cultures of each strain and their respective Δ*pqsE* mutant were diluted to an initial OD_{600} of 0.05, in King's A medium. A volume of 200 µL from each suspension was dispensed in triplicates into sterile 100-well honeycomb microplates and incubated at 37°C in a BioScreen C system (Growth Curves, USA). $OD_{400\text{-}520}$ was determined hourly over 16 hours. Error bars represent standard deviations of the triplicate measurements.

Table S4 : Relative concentration of PCA (mg/mg proteins) for *P. aeruginosa* strains and their Δ*pqsE* mutants. For every WT and Δ*pqsE* mutant, the average concentration of PCA (mg/mg proteins) and the standard deviation of 3 replicates are shown at two timepoints (6 and 24h). Statistical significance (*) between the WT strains and their respective Δ*pqsE* mutants was determined using the Holm-Sidak method, with a significance threshold (*p-value*) of 0.05 (GraphPad Prism). Log2-transformed data were used to generate the heatmap in Fig. 2A. A small pseudocount of 1 was added when the mutant's production or expression was zero to enable log transformation, and these last values were capped at -7.

Strains	6h								24h							
	WT		Δ*pqsE*		*p-value*	*	Δ*pqsE* / WT	Log2	WT		Δ*pqsE*		*p-value*	*	Δ*pqsE* / WT	Log2
	Average	St dev	Average	St dev					Average	St dev	Average	St dev				
**PA14**	6.21E-04	3.42E-04	1.66E-05	1.01E-05	0.044	*	0.027	-3.62	4.75E-04	6.42E-05	9.66E-06	7.44E-07	2.32E-04	*	0.020	-3.89
**PAO1**	7.80E-04	3.34E-04	9.13E-05	1.86E-05	0.023	*	0.117	-2.15	2.66E-04	8.14E-05	3.82E-05	1.25E-06	0.008	*	0.144	-1.94
**39016**	2.29E-04	3.67E-05	8.92E-05	1.06E-05	0.003	*	0.390	-0.940	1.97E-04	3.78E-05	4.95E-05	1.22E-05	0.003	*	0.251	-1.38
**A22**	2.03E-04	7.84E-05	1.10E-04	1.94E-05	0.119	ns	1.00	0.00	5.62E-05	4.90E-05	2.47E-05	2.85E-06	0.328	ns	0.439	0.00
**E90**	2.37E-04	6.60E-05	1.56E-05	2.68E-06	0.004	*	0.066	-2.72	1.90E-04	3.15E-05	1.82E-06	5.82E-07	4.90E-04	*	0.010	-4.65
**JJ692**	7.19E-06	2.71E-06	0.00	0.00	0.010	*	0.00	-7.00	1.31E-04	2.22E-05	4.13E-06	8.62E-07	5.88E-04	*	0.032	-3.46
**PA-CL512**	4.79E-05	1.75E-05	4.37E-06	5.49E-07	0.013	*	0.091	-2.39	2.63E-04	1.41E-04	2.21E-06	7.01E-07	0.032	*	0.008	-4.78
**PA-CL513**	3.75E-04	1.53E-04	4.87E-05	2.08E-05	0.022	*	0.130	-2.04	2.64E-04	2.51E-05	1.36E-05	9.13E-07	6.66E-05	*	0.051	-2.97
**PA-CL521b**	8.57E-04	1.54E-04	3.49E-04	7.97E-05	0.007	*	0.407	-0.900	3.46E-04	6.21E-05	3.01E-05	6.83E-06	0.001	*	0.087	-2.44
**PA-W9**	1.63E-04	7.26E-05	0.00	0.00	0.018	*	0.00	-7.00	4.77E-04	2.20E-04	7.72E-06	2.55E-06	0.021	*	0.016	-4.12
**PG201**	1.07E-04	1.42E-05	4.18E-05	1.03E-05	0.003	*	0.391	-0.940	6.91E-05	1.29E-05	4.42E-05	1.02E-05	0.059	ns	1.00	0.00
**SMC1596**	3.65E-04	1.44E-04	3.79E-05	1.05E-05	0.017	*	0.104	-2.27	1.94E-04	5.13E-05	2.72E-05	9.76E-06	0.023	*	0.140	-1.97

Legend: St dev; Standard deviation, *; Significant difference (*p-value* < 0.05), ns; non-significant difference

Table S5: Relative concentration of pyocyanin (mg/mg proteins) for *P. aeruginosa* strains and their Δ*pqsE* mutants. For every WT and Δ*pqsE* mutant, the average concentration of pyocyanin (mg/mg proteins) and the standard deviation of 3 replicates are shown at two timepoints (6 and 24h). Statistical significance (*) between the WT strains and their respective Δ*pqsE* mutants was determined using the Holm-Sidak method, with a significance threshold (*p-value*) of 0.05 (GraphPad Prism). Log2-transformed data were used to generate the heatmap in Fig. 2A. A small pseudocount of 1 was added when the mutant's production or expression was zero to enable log transformation, and these last values were capped at -7.

Strains	6h								24h							
	WT		Δ*pqsE*		*p-value*	*	Δ*pqsE* / WT	Log2	WT		Δ*pqsE*		*p-value*	*	Δ*pqsE* / WT	Log2
	Average	St dev	Average	St dev					Average	St dev	Average	St dev				
**PA14**	1.34E-04	2.99E-05	5.26E-06	2.94E-06	0.004	*	0.039	-3.24	1.19E-04	1.69E-05	3.34E-06	2.26E-07	2.94E-04	*	0.028	-3.57
**PAO1**	3.08E-04	1.53E-04	3.5E-05	4.30E-06	0.037	*	0.113	-2.18	1.36E-04	4.96E-05	1.64E-05	1.02E-06	0.014	*	0.120	-2.12
**39016**	4.89E-05	8.53E-06	1.85E-05	1.98E-06	0.004	*	0.379	-0.970	5.90E-05	1.11E-05	1.24E-05	3.03E-06	0.002	*	0.210	-1.56
**A22**	6.91E-05	2.30E-05	3.40E-05	5.26E-06	0.062	ns	1.00	0.00	4.22E-05	3.22E-05	2.32E-05	1.53E-06	0.365	ns	1.00	0.00
**E90**	9.49E-05	2.46E-05	4.99E-06	1.20E-06	0.003	*	0.053	-2.95	2.04E-04	4.10E-05	8.95E-06	3.85E-06	0.001	*	0.044	-3.13
**JJ692**	1.51E-06	5.75E-07	0.00	0.00	0.010	*	0.00	-7.00	2.42E-04	4.75E-05	1.00E-06	3.45E-07	0.001	*	0.004	-5.49
**PA-CL512**	4.64E-05	3.00E-06	1.63E-06	1.82E-07	1.35E-05	*	0.035	-3.35	3.62E-04	1.44E-04	1.23E-05	2.61E-06	0.014	*	0.034	-3.38
**PA-CL513**	1.36E-04	3.28E-05	1.36E-05	5.36E-06	0.003	*	0.100	-2.30	1.15E-04	1.29E-05	5.12E-06	4.90E-07	1.22E-04	*	0.044	-3.11
**PA-CL521b**	2.68E-04	5.02E-05	1.08E-04	2.30E-05	0.007	*	0.402	-0.911	2.56E-04	4.56E-05	4.93E-05	1.57E-05	0.002	*	0.193	-1.65
**PA-W9**	4.16E-05	1.70E-05	0.00	0.00	0.013	*	0.00	-7.00	3.53E-04	1.53E-04	7.39E-07	6.58E-07	0.016	*	0.002	-6.17
**PG201**	5.33E-05	9.57E-06	1.62E-05	4.10E-06	0.004	*	0.305	-1.19	5.42E-05	7.81E-06	3.99E-05	8.18E-06	0.094	ns	1.00	0.00
**SMC1596**	6.7E-05	2.77E-05	7.2E-06	5.27E-07	0.020	*	0.108	-2.23	3.23E-05	8.80E-06	8.00E-06	3.20E-06	0.037	*	0.247	-1.40

Legend: St dev; Standard deviation, *; Significant difference (*p-value* < 0.05), ns; non-significant difference

Table S6: Relative concentration of rhamnolipids (mg/mg proteins) for *P. aeruginosa* strains and their Δ*pqsE* mutants. For every WT and Δ*pqsE* mutant, the average concentration of rhamnolipids (mg/mg proteins) and the standard deviation of 3 replicates are shown after 24h of growth in King's A medium. Statistical significance (*) between the WT strains and their respective Δ*pqsE* mutants was determined using the Holm-Sidak method, with a significance threshold (*p-value*) of 0.05 (GraphPad Prism). When a significant difference was found, the ratio of the production of the *pqsE* mutant and the WT strain was calculated, and log2-transformed data were used to generate the heatmap in Fig. 2B.

Strains	Mono-rhamnolipids (Rha-C10-C10)								Di-rhamnolipids (Rha-Rha-C10-C10)							
	WT		Δ*pqsE*		*p-value*	*	Δ*pqsE* / WT	Log2	WT		Δ*pqsE*		*p-value*	*	Δ*pqsE* / WT	Log2
	Average	St dev	Average	St dev					Average	St dev	Average	St dev				
**PA14**	0.042	0.009	0.036	0.005	0.298	ns	1.00	0.00	0.121	0.018	0.111	0.011	0.467	ns	1.00	0.00
**PAO1**	0.017	0.004	0.014	0.002	0.312	ns	1.00	0.00	0.118	0.015	0.127	0.024	0.609	ns	1.00	0.00
**39016**	0.041	0.011	0.042	0.006	0.860	ns	1.00	0.00	0.136	0.036	0.168	0.032	0.309	ns	1.00	0.00
**A22**	0.050	0.007	0.059	0.011	0.306	ns	1.00	0.00	0.106	0.023	0.125	0.022	0.364	ns	1.00	0.00
**E90**	0.036	0.007	0.018	0.004	0.014	*	0.486	-0.72	0.199	0.038	0.104	0.005	0.013	*	0.524	-0.646
**JJ692**	0.013	0.002	0.005	0.001	0.002	*	0.355	-1.04	0.092	0.007	0.037	0.006	4.38E-04	*	0.401	-0.914
**PA-CL512**	0.064	0.011	0.009	0.002	0.001	*	0.134	-2.01	0.187	0.027	0.029	0.008	6.19E-04	*	0.156	-1.86
**PA-CL513**	0.041	0.007	0.031	0.007	0.204	ns	1.00	0.00	0.115	0.026	0.117	0.005	0.928	ns	1.00	0.00
**PA-CL521b**	0.051	0.006	0.053	0.007	0.748	ns	1.00	0.00	0.123	0.007	0.118	0.028	0.782	ns	1.00	0.00
**PA-W9**	0.035	0.005	0.027	0.004	0.049	*	0.773	-0.258	0.124	0.020	0.103	0.013	0.213	ns	1.00	0.00
**PG201**	0.023	0.004	0.022	0.004	0.707	ns	1.00	0.00	0.123	0.035	0.114	0.018	0.709	ns	1.00	0.00
**SMC1596**	0.034	0.003	0.038	0.005	0.347	ns	1.00	0.00	0.101	0.014	0.133	0.036	0.229	ns	1.00	0.00

Legend: St dev; Standard deviation, *; Significant difference (*p-value* < 0.05), ns; non-significant difference

Table S7 : Biofilm production (OD_{550}) for *P. aeruginosa* strains and their Δ*pqsE* mutants. Biofilm production was measured using crystal violet dye, as described before (14). For every WT and Δ*pqsE* mutant, the average production of biofilms (OD_{550}) and the standard deviation of 5 replicates are shown after 24h and 48 hours of growth. Statistical significance (*) between the WT strains and their respective Δ*pqsE* mutants was determined using the Holm-Sidak method, with a significance threshold (*p-value*) of 0.05 (GraphPad Prism). When a significant difference was found, the ratio of the production of the Δ*pqsE* mutant and the WT strain was calculated, and log2-transformed data were used to generate the heatmap in Fig. 4.

Strains	24h								48h							
	WT		Δ*pqsE*		*p-value*	*	Δ*pqsE* / WT	Log2	WT		Δ*pqsE*		*p-value*	*	Δ*pqsE* / WT	Log2
	Average	St dev	Average	St dev					Average	St dev	Average	St dev				
**PA14**	0.302	0.088	0.143	0.003	0.004	*	0.473	-0.749	0.225	0.009	0.074	0.013	1.99E-08	*	0.328	-1.12
**PAO1**	0.144	0.019	0.298	0.068	0.002	*	2.06	0.725	0.144	0.019	0.298	0.068	0.001	*	2.06	0.725
**39016**	0.092	0.028	0.131	0.011	0.021	*	1.42	0.352	0.021	0.006	0.060	0.016	0.001	*	2.87	1.05
**A22**	0.357	0.060	0.284	0.023	0.065	ns	1.00	0.00	0.033	0.006	0.042	0.013	0.191	ns	1.00	0.00
**E90**	0.095	0.020	0.127	0.050	0.212	ns	1.00	0.00	0.189	0.065	0.723	0.079	2.64E-06	*	3.82	1.34
**JJ692**	0.053	0.004	0.101	0.025	0.010	*	1.91	0.649	0.030	0.009	0.049	0.012	0.027	*	1.60	0.469
**PA-CL512**	0.156	0.020	0.193	0.015	0.010	*	1.24	0.212	0.044	0.014	0.061	0.012	0.075	ns	1.00	0.00
**PA-CL513**	0.104	0.005	0.180	0.018	1.84E-05	*	1.73	0.546	0.012	0.002	0.049	0.008	7.79E-06	*	4.08	1.41
**PA-CL521b**	0.302	0.045	0.207	0.019	0.002	*	0.685	-0.378	0.095	0.019	0.128	0.023	0.037	*	1.35	0.301
**PA-W9**	0.083	0.0102	0.062	0.006	0.004	*	0.748	-0.291	0.034	0.004	0.049	0.015	0.068	ns	1.00	0.00
**PG201**	0.087	0.006	0.079	0.011	0.213	ns	1.00	0.00	0.205	0.009	0.021	0.003	8.59E-11	*	0.101	-2.29
**SMC1596**	0.294	0.091	0.097	0.019	0.005	*	0.329	-1.11	0.170	0.026	0.124	0.018	0.011	*	0.727	-0.319

Table S8: Relative concentration of HHQ (mg/mg proteins) for *P. aeruginosa* strains and their Δ*pqsE* mutants. For every WT and Δ*pqsE* mutant, the average concentration of HHQ (mg/mg proteins) and the standard deviation of 3 replicates are shown at two timepoints (6 and 24h). Statistical significance (*) between the WT strains and their respective Δ*pqsE* mutants was determined using the Holm-Sidak method, with a significance threshold (*p-value*) of 0.05 (GraphPad Prism). Log2-transformed data were used to generate the heatmap in Figure 2. A small pseudocount of 1 was added when the mutant's production or expression was zero to enable log transformation, and these last values were capped at -5.

Strains	6h								24h							
	WT		Δ*pqsE*		*p-value*	*	Δ*pqsE* / WT	Log2	WT		Δ*pqsE*		*p-value*	*	Δ*pqsE* / WT	Log2
	Average	St dev	Average	St dev					Average	St dev	Average	St dev				
**PA14**	3.39E-04	2.43E-04	3.14E-04	1.85E-04	0.903	ns	1.00	0.00	5.74E-05	1.29E-05	1.19E-04	1.29E-05	0.035	*	2.07	0.728
**PAO1**	8.10E-04	3.30E-04	4.36E-04	3.85E-05	0.123	ns	1.00	0.00	7.57E-05	2.10E-05	1.12E-04	5.71E-06	0.044	*	1.48	0.394
**39016**	1.27E-04	4.33E-05	6.12E-04	7.36E-05	0.001	*	4.80	1.57	2.26E-05	6.63E-06	5.83E-05	1.55E-05	0.021	*	2.58	0.946
**E90**	2.29E-03	2.97E-04	2.17E-05	5.98E-06	1.90E-04	*	0.009	-4.66	2.93E-04	4.66E-05	1.84E-05	1.64E-05	0.001	*	0.063	-2.77
**JJ692**	1.24E-03	1.39E-04	2.31E-05	1.46E-05	1.14E-04	*	0.019	-3.98	7.89E-04	2.57E-04	9.32E-05	2.79E-05	0.010	*	0.118	-2.14
**PA-CL512**	2.21E-03	5.28E-05	7.90E-04	8.67E-05	1.71E-05	*	0.357	-1.03	2.02E-03	5.95E-04	7.48E-04	7.04E-05	0.021	*	0.370	-0.993
**PA-CL513**	4.60E-04	9.71E-05	9.50E-04	4.11E-04	0.115	ns	1.00	0.00	2.87E-05	1.05E-06	5.70E-05	1.15E-05	0.013	*	1.99	0.686
**PA-CL521b**	2.85E-03	1.29E-04	1.92E-05	1.92E-05	2.92E-06	*	0.007	-5.00	3.01E-04	3.84E-05	4.74E-05	6.02E-05	0.004	*	0.157	-1.85
**PA-W9**	2.02E-03	7.04E-04	1.20E-04	3.28E-05	0.009	*	0.059	-2.82	3.41E-04	1.59E-04	7.38E-06	1.98E-06	0.022	*	0.022	-3.83
**PG201**	8.34E-05	2.35E-06	0.00	0.00	4.2E-07	*	0.00	-5.00	1.01E-05	9.40E-07	0.00E+00	0.00E+00	4.88E-05	*	0.00	-5.00
**SMC1596**	2.76E-04	6.85E-05	2.70E-04	7.10E-05	0.913	ns	1.00	0.00	4.37E-05	1.38E-05	4.18E-05	1.73E-05	0.902	ns	1.00	0.00

Legend: St dev; Standard deviation, *; Significant difference (*p-value* < 0.05), ns; non-significant difference

Table S9: Relative concentration of PQS (mg/mg proteins) for *P. aeruginosa* strains and their Δ*pqsE* mutants. For every WT and Δ*pqsE* mutant, the average concentration of PQS (mg/mg proteins) and the standard deviation of 3 replicates are shown at two timepoints (6 and 24h). Statistical significance (*) between the WT strains and their respective Δ*pqsE* mutants was determined using the Holm-Sidak method, with a significance threshold (*p-value*) of 0.05 (GraphPad Prism). Log2-transformed data were used to generate the heatmap in Figure 2. A small pseudocount of 1 was added when the mutant's production or expression was zero to enable log transformation, and these last values were capped at -5.

Strains	6h								24h							
	WT		Δ*pqsE*		*p-value*	*	Δ*pqsE* / WT	Log2	WT		Δ*pqsE*		*p-value*	*	Δ*pqsE* / WT	Log2
	Average	St dev	Average	St dev					Average	St dev	Average	St dev				
**PA14**	1.48E-03	1.14E-03	6.52E-04	2.76E-04	0.282	ns	1.00	0.00	1.50E-03	2.35E-04	3.07E-03	2.35E-04	0.006	*	2.05	0.717
**PAO1**	8.10E-04	0.000330	4.36E-04	3.85E-05	0.407	ns	1.00	0.00	2.25E-03	5.96E-04	2.23E-03	2.91E-04	0.952	ns	1.00	0.00
**39016**	1.93E-04	7.18E-05	5.96E-04	1.25E-04	0.008	*	3.10	1.13	4.28E-04	1.20E-04	1.14E-03	5.01E-04	0.070	ns	1.00	0.00
**E90**	6.82E-04	1.08E-04	0.00	0.00	3.97E-04	*	0.00	-5.00	5.70E-04	3.58E-04	6.57E-05	4.66E-05	0.073	ns	1.00	0.00
**JJ692**	1.48E-04	3.89E-05	0.00	0.00	0.003	*	0.00	-5.00	1.86E-04	3.20E-05	2.46E-05	1.03E-05	0.001	*	0.132	-2.03
**PA-CL512**	4.04E-04	1.02E-04	1.67E-04	3.25E-05	0.019	*	0.414	-0.882	3.51E-03	1.32E-03	1.15E-03	8.58E-05	0.037	*	0.326	-1.12
**PA-CL513**	1.25E-03	2.63E-04	1.44E-03	1.07E-03	0.777	ns	1.00	0.00	1.32E-03	8.44E-05	2.17E-03	4.00E-04	0.023	*	1.65	0.499
**PA-CL521b**	3.48E-04	3.59E-05	0.00	0.00	7.36E-05	*	0.00	-5.00	3.68E-03	1.30E-04	8.81E-04	2.77E-04	9.33E-05	*	0.240	-1.43
**PA-W9**	6.04E-04	1.75E-04	1.44E-04	5.90E-05	0.012	*	0.239	-1.43	3.23E-03	1.77E-03	1.64E-04	2.20E-05	0.040	*	0.051	-2.98
**PG201**	0.00	0.00	0.00	0.00	N/A	N/A	N/A	N/A	0.00	0.00	0.00	0.00	N/A	N/A	N/A	N/A
**SMC1596**	1.56E-03	0.0006	0.000375	0.0001	0.022	*	0.240	-1.43	9.76E-04	2.53E-04	5.93E-04	3.22E-04	0.229	ns	1.00	0.00

Legend: St dev; Standard deviation, *; Significant difference (*p-value* < 0.05), ns; non-significant difference, N/A ; Non-applicable (i.e the strain does not produce PQS)

Table S10 : List of plasmids used in this study

Plasmid	Characteristics	Source
pEX18ApΔ*pqsE*	Suicide vector containing a 570-bp deletion in the *pqsE* gene, *sacB,* Amp^R	(2)
pEX18Gm	Suicide vector, *sacB*, Gm^R	(15)
pMT01	pEX18GmΔ*pqsE;* Suicide vector containing a 570-bp deletion in the *pqsE* gene, *sacB,* Gm^R	This study

Table S11: List of primers used in this study

Primer		Sequence	Function	Source
pqsE_verif	F	CTGGATGATGACCTGTGCCT	Δ*pqsE* mutant verification	This study
	R	CATCGAGGAAGAAGCGGTAC		
pqsE_amp	F	ATGTTGAGGCTTTCGGCTC	Amplification of the *pqsE* gene	This study
	R	AATGGATGTCCCGCCTCAGT		
pqsE_qRT	F	CTGGTTGAAGGAGGGATCAG	Amplification of *pqsE* for RT-qPCR	This study
	R	GTCGTAGTGCTTGTGGGTGA		
qnadB	F	CTACCTGGACATCAGCCACA	Amplification of *nadB* for RT-qPCR	(16)
	R	GGTAATGTCGATGCCGAAG		

References

1. Rahme LG, Stevens EJ, Wolfort SF, Shao J, Tompkins RG, Ausubel FM. 1995. Common virulence factors for bacterial pathogenicity in plants and animals. Science 268:1899-902.
2. Déziel E, Lépine F, Milot S, He J, Mindrinos MN, Tompkins RG, Rahme LG. 2004. Analysis of *Pseudomonas aeruginosa* 4-hydroxy-2-alkylquinolines (HAQs) reveals a role for 4-hydroxy-2-heptylquinoline in cell-to-cell communication. Proc Natl Acad Sci U S A 101:1339-44.
3. Dulcey CE, Dekimpe V, Fauvelle DA, Milot S, Groleau MC, Doucet N, Rahme LG, Lépine F, Déziel E. 2013. The end of an old hypothesis: the *pseudomonas* signaling molecules 4-hydroxy-2-alkylquinolines derive from fatty acids, not 3-ketofatty acids. Chem Biol 20:1481-91.
4. Rampioni G, Pustelny C, Fletcher MP, Wright VJ, Bruce M, Rumbaugh KP, Heeb S, Camara M, Williams P. 2010. Transcriptomic analysis reveals a global alkyl-quinolone-independent regulatory role for PqsE in facilitating the environmental adaptation of *Pseudomonas aeruginosa* to plant and animal hosts. Environ Microbiol 12:1659-73.
5. Freschi L, Jeukens J, Kukavica-Ibrulj I, Boyle B, Dupont MJ, Laroche J, Larose S, Maaroufi H, Fothergill JL, Moore M, Winsor GL, Aaron SD, Barbeau J, Bell SC, Burns JL, Camara M, Cantin A, Charette SJ, Dewar K, Déziel E, Grimwood K, Hancock RE, Harrison JJ, Heeb S, Jelsbak L, Jia B, Kenna DT, Kidd TJ, Klockgether J, Lam JS, Lamont IL, Lewenza S, Loman N, Malouin F, Manos J, McArthur AG, McKeown J, Milot J, Naghra H, Nguyen D, Pereira SK, Perron GG, Pirnay JP, Rainey PB, Rousseau S, Santos PM, Stephenson A, Taylor V, Turton JF, Waglechner N, et al. 2015. Clinical utilization of genomics data produced by the international *Pseudomonas aeruginosa* consortium. Front Microbiol 6:1036.
6. Pirnay JP, Bilocq F, Pot B, Cornelis P, Zizi M, Van Eldere J, Deschaght P, Vaneechoutte M, Jennes S, Pitt T, De Vos D. 2009. *Pseudomonas aeruginosa* population structure revisited. PLoS One 4:e7740.

7. Feltner JB, Wolter DJ, Pope CE, Groleau MC, Smalley NE, Greenberg EP, Mayer-Hamblett N, Burns J, Déziel E, Hoffman LR, Dandekar AA. 2016. LasR Variant Cystic Fibrosis Isolates Reveal an Adaptable Quorum-Sensing Hierarchy in *Pseudomonas aeruginosa*. mBio 7:e01513-16.
8. Wolfgang MC, Kulasekara BR, Liang X, Boyd D, Wu K, Yang Q, Miyada CG, Lory S. 2003. Conservation of genome content and virulence determinants among clinical and environmental isolates of *Pseudomonas aeruginosa*. Proc Natl Acad Sci U S A 100:8484-9.
9. Yu Q, Griffin EF, Moreau-Marquis S, Schwartzman JD, Stanton BA, O'Toole GA. 2012. In vitro evaluation of tobramycin and aztreonam versus *Pseudomonas aeruginosa* biofilms on cystic fibrosis-derived human airway epithelial cells. J Antimicrob Chemother 67:2673-81.
10. Lalancette C, Charron D, Laferriere C, Dolce P, Déziel E, Prévost M, Bédard E. 2017. Hospital Drains as Reservoirs of *Pseudomonas aeruginosa*: Multiple-Locus Variable-Number of Tandem Repeats Analysis Genotypes Recovered from Faucets, Sink Surfaces and Patients. Pathogens 6.
11. Guerra-Santos L, Käppeli O, Fiechter A. 1984. *Pseudomonas aeruginosa* biosurfactant production in continuous culture with glucose as carbon source. Appl Environ Microbiol 48:301-305.
12. Katoh K, Rozewicki J, Yamada KD. 2019. MAFFT online service: multiple sequence alignment, interactive sequence choice and visualization. Brief Bioinform 20:1160-1166.
13. Seemann T. 2014. Prokka: rapid prokaryotic genome annotation. Bioinformatics 30:2068-9.
14. O'Toole GA. 2011. Microtiter Dish Biofilm Formation Assay. J Vis Exp 30:e2437.
15. Hoang TT, Karkhoff-Schweizer RR, Kutchma AJ, Schweizer HP. 1998. A broad-host-range Flp-FRT recombination system for site-specific excision of chromosomally-located DNA sequences: application for isolation of unmarked *Pseudomonas aeruginosa* mutants. Gene 212:77-86.
16. Tremblay J, Déziel E. 2010. Gene expression in *Pseudomonas aeruginosa* swarming motility. BMC Genomics 11:587.
